# A Genome-wide screen identifies frequently methylated genes in haematological and epithelial cancers

**DOI:** 10.1186/1476-4598-9-44

**Published:** 2010-02-25

**Authors:** Thomas Dunwell, Luke Hesson, Tibor A Rauch, Lihui Wang, Richard E Clark, Ashraf Dallol, Dean Gentle, Daniel Catchpoole, Eamonn R Maher, Gerd P Pfeifer, Farida Latif

**Affiliations:** 1 Department of Medical and Molecular Genetics, School of Clinical and Experimental Medicine, College of Medical and Dental Sciences, University of Birmingham, Birmingham B15 2TT, UK; 2 Beckman Research Institute, City of Hope, 1500 E. Duarte Road, Duarte, CA 91010.USA; 3 Department of Haematology, Royal Liverpool University Hospital, Liverpool, L7 8XP. UK; 4 The Children's Hospital at Westmead, Locked Bag 4001, Westmead, NSW, 2145. Australia

## Abstract

**Background:**

Genetic as well as epigenetic alterations are a hallmark of both epithelial and haematological malignancies. High throughput screens are required to identify epigenetic markers that can be useful for diagnostic and prognostic purposes across malignancies.

**Results:**

Here we report for the first time the use of the MIRA assay (methylated CpG island recovery assay) in combination with genome-wide CpG island arrays to identify epigenetic molecular markers in childhood acute lymphoblastic leukemia (ALL) on a genome-wide scale. We identified 30 genes demonstrating methylation frequencies of ≥25% in childhood ALL, nine genes showed significantly different methylation frequencies in B vs T-ALL. For majority of the genes expression could be restored in methylated leukemia lines after treatment with 5-azaDC. Forty-four percent of the genes represent targets of the polycomb complex. In chronic myeloid leukemia (CML) two of the genes, (*TFAP2A *and *EBF2)*, demonstrated increased methylation in blast crisis compared to chronic phase (P < 0.05). Furthermore hypermethylation of an autophagy related gene *ATG16L2 *was associated with poorer prognosis in terms of molecular response to Imatinib treatment. Lastly we demonstrated that ten of these genes were also frequently methylated in common epithelial cancers.

**Conclusion:**

In summary we have identified a large number of genes showing frequent methylation in childhood ALL, methylation status of two of these genes is associated with advanced disease in CML and methylation status of another gene is associated with prognosis. In addition a subset of these genes may act as epigenetic markers across hematological malignancies as well as common epithelial cancers.

## Background

The role of aberrant DNA methylation in the development of cancer is well recognized and documented. Tumor suppressor gene (TSG) inactivation by promoter region CpG island hypermethylation occurs in almost all cancer types and is an important mechanism of gene silencing in cancer. Unlike genetic changes in cancer, epigenetic changes are potentially reversible. Epigenetic therapy is a rapidly expanding field and a number of drugs that alter the epigenetic profiles of cancer cells are already in clinical trails. Two hypomethylating agents, 5-azacitidine (Vidaza) and 5-aza-2'-deoxycytidine (Decitabine) are currently in use and are approved therapies for myelodysplastic syndrome [1,2].

*RASSF1A *TSG is a classic example of a gene that is frequently methylated in the majority of adult and childhood cancers including epithelial and blood borne cancers [3]. The *RASSF1 *family of genes now includes 10 members (*RASSF1-10*). We have recently described an epigenetic profile of the *RASSF1-10 *genes in childhood acute lymphoblastic leukemia (ALL) [4]. Our novel findings indicate that *RASSF6 *and *RASSF10 *are frequently and specifically methylated in ALL in contrast to *RASSF1A *which is frequently methylated in epithelial cancers including lung, breast and kidney cancer but shows low frequency of methylation in childhood ALL. In addition we have demonstrated that methylation frequencies differ between B and T-ALL. Whilst *RASSF6 *is methylated in the majority of (94%) B-ALL and less than half (41%) of T-ALL. *RASSF10 *methylation frequency shows the opposite trend (16% in B-ALL vs 88% in T-ALL). Using a chromosome 3 Not1 array hybridization approach, we recently identified a number of genes frequently methylated in acute lymphoblastic leukemia [5].

We have now used a recently developed high throughput approach, methylated CpG island recovery assay (MIRA) in combination with genome-wide CpG island arrays to identify epigenetic molecular markers in childhood ALL on a genome-wide scale. MIRA (methylated-CpG island recovery assay) is based on the high affinity of the methyl-CpG binding protein complex MBD2/MBD3L1 to methylated DNA [6-8]. MIRA assay has several advantages for use as a tool for comprehensive analysis of DNA methylation patterns, for example it does not depend on having specific methylation-sensitive restriction sites in the target sequence, it does not depend on use of antibodies against 5-methylcytosine, and the ease of preparation of the recombinant GST tagged MBD2 and MBD3L1.

## Results

### Identification of methylated genes in childhood ALL on a genome-wide based platform

We used the sensitive MIRA method in combination with genome-wide CpG island arrays to identify frequently methylated genes in childhood ALL. A total of five T-ALL samples were selected for analysis using the MIRA assay followed by methylation analysis using a Agilent, Human CpG Island Chip on chip 244 k array. The five selected samples were all males to avoid sex based variation and ranged in age at diagnosis from 5.37 to 12.62 years, there were also no reported translocations in the samples to avoid specific translocation derived methylation. Age matched peripheral blood lymphocytes from normal healthy individuals were used as controls in the assay.

**Figure 1:**
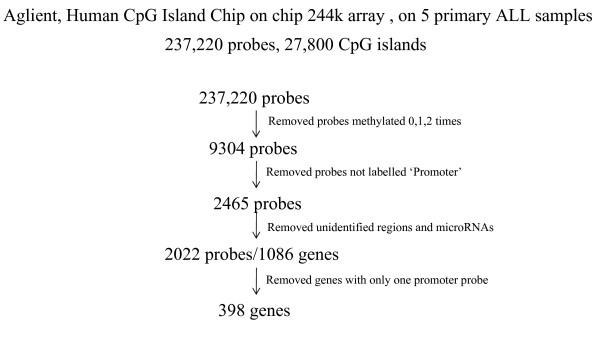
**Candidate gene selection**. This schematic details the criteria used in the microarray data analysis for the selection of the short list of 398 genes. The short list, only contains genes which had two or more probes labelled as 'promoter' which were methyled in at least three of the five primary samples analysed, this list does not contain any microRNAs or unidentified genes/chromosomal regions.

The Agilent array is advertised with 237,220 probes covering 27,800 CpG Islands. A series of criteria were decided upon to reduce the number of results to a selection of gene-associated CpG islands that would be assayed for hypermethylation. The total combined data set for the five primary samples contained 199,416 CpG island probes covering 14,020 genes. The first level of data filtering involved removal of all array probes that only showed methylation (≥ 3 fold enrichment of the methylated fraction in leukemia samples versus control samples) in 0, 1 or 2 out of the five samples analysed, this removed a total of 190,112 probes. The remaining 9304 probes covered 3061 genes, 43 hypothetical proteins, 31 chromosomal loci, 127 predicted open reading frames, 1728 chromosomal regions and 25 microRNAs. Many of the CpG islands were represented by 2 or more probes and therefore is an over representation of the number of CpG Islands. To further enrich the results for true positive results that would result in identification of novel hypermethylated genes, all probes not labelled by Agilent as 'promoter' were removed. This removed all probes labelled as 'divergent promoter', 'downstream' and 'inside' leaving 2465 probes. Following this all remaining hypothetical proteins, chromosomal loci, predicted open reading frames, chromosomal regions and microRNAs were removed, leaving only known genes. This left 2022 probes covering 1086 genes. The final step to enrich the data for genes showing only the highest level of methylation involved removal of all genes which only had a single methylated probe labelled as 'promoter'. This resulted in a final list of 398 genes. Lastly functional annotation was carried out to determine likely roles in tumorigenesis. Based on this functional annotation candidate genes were selected from this final short list and methylation status of their CpG island's were investigated. A total of 54 genes, and 62 associated CpG islands (due to multiple isoforms) were selected for analysis (Figure 1).

### Validation of gene methylation in ALL samples

**Figure 2:**
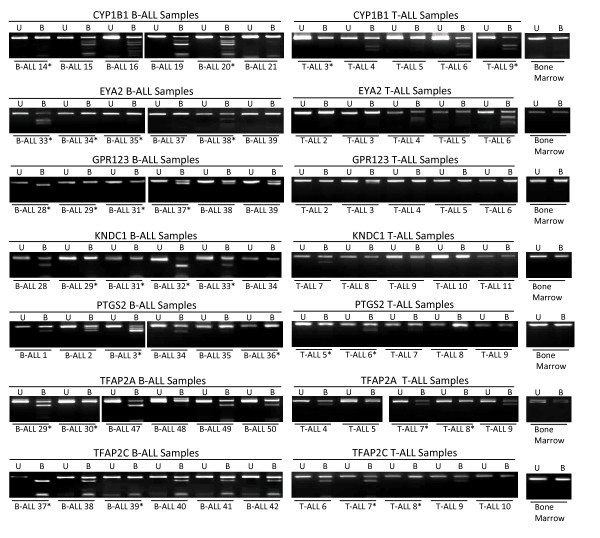
**Methylation analysis in B and T-ALL**. Representative combined bisulfite restriction analysis (COBRA) results for primary T-ALL and B-ALL samples and control bone marrow. No methylation was detected in any of the control bone marrow samples. U = undigested PCR product, B = BstUI digested PCR product. The samples labelled with * correspond to those ALL samples for which methylation was assessed by cloning and sequencing.

We undertook COBRA (combined bisulfite restriction analysis) analysis to confirm and extend the methylation profile of the above genes in a series of B (n = 52) and T (n = 12) -childhood acute lymphoblastic leukemia samples (Figure 2) and in a series of leukemia cell lines (n = 12) (see Additional file 1). We used DNA from age matched normal healthy blood lymphocytes and bone marrow (n = 10) as controls. Where possible COBRA primers were designed to encompass the transcription start site within the CpG island or to within 500bp of the transcription start site. *BstUI *and *TaqI *digest was used for digestion of COBRA PCR products. Amongst the 54 genes analysed 30 (56%) genes demonstrated frequent methylation (≥ 25%) in primary B or T ALL or both subtypes as well as leukemia cell lines, with the exception of FAT1, DUSP4 and POU4F1 that demonstrated lower methylation frequency in leukemia cell lines compared to primary ALL samples (Table 1). Two further genes showed methylation frequency of 23% and are included in table 1. The majority of the above genes (n = 29) were unmethylated in DNA from control peripheral blood lymphocytes and bone marrow samples, 2 genes (*EBF2*, *HLA-F*) demonstrated some methylation in 1/10 control samples and one gene (*MYO10*) showed methylation in 1/8 control samples. Nine genes demonstrated differential methylation that was statistically significant in B vs T-ALL (P < 0.05). Five genes were significantly more methylated in B-ALL compared to T-ALL (*BARHL2, CYP1B1, FAT1, PTGS2, TSHZ3*), whilst four genes showed more methylation in T compared to B-ALL (*BMP2, MYO10, NR4A2, TCF2*). Future studies using larger sample sets would be required to validate the differential methylation patterns seen above. Another 6 (*TAC1, HMX2, HLA-G, VSNL1, PAX7, PAX9*) genes demonstrated frequent methylation in leukemia cell lines but were also frequently methylated in DNA from healthy bone marrow and a further 2 genes showed cancer specific methylation from analysis of leukemia cell lines and healthy bone marrow and blood DNA but showed either no or very low frequency of methylation in primary ALL samples (*TNFAIP1, TLR2*). Whilst 14 genes showed no or very low frequency of methylation in leukemia cell lines, hence these were not analysed any further (see Additional file 2).

**Table 1:** Summary of the promoter hypermethylation frequencies and expression analysis of candidate genes in ALL.

Gene	Cell lines	T-ALL (%)	B-ALL (%)	PB/BM controls	Expression upregulation after 5azaDC
ARHGAP20	6/10	4/12 (33)	33/51 (65)	0/8	YES

ATG16L2	3/7	5/12 (42)	14/52 (27)	0/8	ND

BARHL2†	5/6	3/12 (25)	39/51 (77)	0/9	YES

BMP2†	6/7	7/12 (58)	2/52 (4)	0/8	YES

CDC14B	5/7	4/12 (33)	21/48 (44)	0/9	YES

CYP1B1†	5/6	5/12 (42)	40/52 (77)	0/10	YES

DUSP4	1/6	3/12 (25)	13/52 (25)	0/6	ND

EBF2	5/6	10/12 (83)	49/52 (94)	1/10	YES

EYA2	5/7	3/12 (25)	15/47 (32)	0/10	YES

FAT1†	1/6	3/12 (25)	34/52 (65)	0/10	YES

FOXF2	4/6	3/12 (25)	15/52 (29)	0/9	YES

GPR123	5/7	4/12 (33)	10/48 (21)	0/9	YES

HLA-F	4/6	4/12 (33)	7/52 (14)	1/10	ND

KNDC1	4/7	1/12 (8)	9/40 (23)	0/9	YES

MYO10†	6/8	6/12 (50)	8/51 (16)	1/8	YES

NKX2-1	5/6	5/12 (42)	32/51 (63)	0/9	YES

N2RE1	6/6	4/12 (33)	19/49 (39)	0/8	YES

NR4A2†	3/7	6/12 (50)	5/51 (10)	0/9	ND

PAX2	6/7	2/10 (20)	15/34 (44)	0/9	YES

PAX6	3/6	6/12 (50)	35/52 (67)	0/10	YES

POU4F1	1/6	5/12 (42)	22/51 (43)	0/8	YES

PRDM12	6/6	6/12 (50)	30/45 (67)	0/9	YES

PTGS2†	4/6	6/12 (50)	48/52 (92)	0/10	YES

SALL3	5/6	4/12 (33)	25/52 (48)	0/9	YES

SSPN	7/9	4/12 (33)	32/52 (62)	0/8	YES

TCF2†	5/6	6/12 (50)	5/52 (10)	0/10	YES

TFAP2A	5/6	5/11 (46)	33/49 (67)	0/10	YES

TFAP2C	2/6	10/12 (83)	43/50 (86)	0/10	YES

TP53I11	3/6	3/12 (25)	8/52 (15)	0/9	ND

TRPC4	5/5	9/12 (75)	48/52 (92)	0/10	YES

TSHZ3†	4/6	0/12 (0)	16/52 (31)	0/10	ND

UBE2C	3/6	2/12 (17)	12/52 (23)	0/10	YES

The table summarises the genes showing a methylation frequency of 25% or above (with the exception of *UBE2C *and *KNDC1*) in either T or B-ALL or both, that were unmethylated or showed much lower frequency of methylation in control blood/bone marrow DNA. Also indicated are the genes showing upregulated expression in methylated leukemia cell lines (but were not upregulated in unmethylated cell lines) after 5-azaDC treatment ('YES' in the expression upregulation after 5-azaDC column). **PB**; peripheral blood lymphocytes from healthy donors. **BM**; bone marrow DNA from healthy donors. **ND**; not determined. †; significantly differential methylation in T-ALL vs B-ALL.

### Cloning and direct sequencing of bisulfite modified DNA

**Figure 3:**
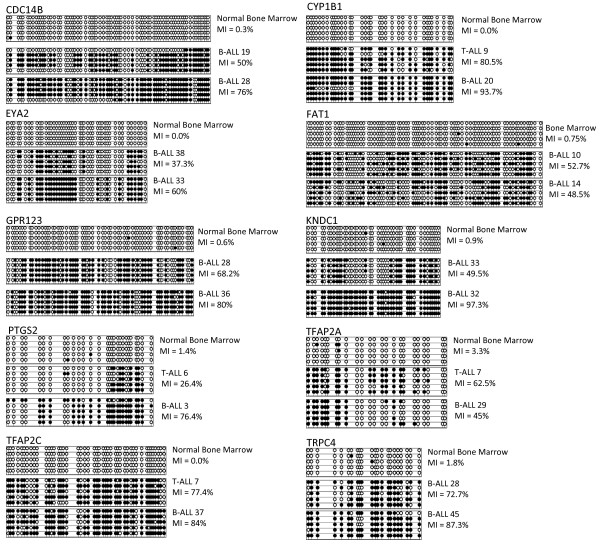
**Methylation assessed by sequencing**. Representative results of cloning and sequencing of ten genes. COBRA PCR products were cloned into PGem T-easy vector and colonies were picked and inserts sequenced. Each line is representative of an individual allele. Each circle represents a single CpG dinucleotide, filled circles represent a methylated CpG dinucleotide whereas clear circles represent an unmethylated CpG dinucleotide. The MI was calculated as a percentage using the equation; number of CpG dinucleotides methylated/total number of CpG dinucleotides sequenced x 100. Sequencing of the DNA from control bone marrow shows no or very infrequent methylation, with MIs all below 3.5%. For each gene two samples for which COBRA analysis detected hypermethylation and two for which it did not were sequenced. The figure shows control bone marrow and two methylated samples for each gene. The sequencing results correlate with the COBRA detected methylation.

To assess the extent of CpG island methylation within the genes showing cancer specific methylation, bisulfite modified DNA from primary ALL samples as well as blood lymphocytes and bone marrow DNA from age matched control samples was cloned and sequenced (Figure 3). As seen in figure 3 healthy control bone marrow DNA samples show either no or very low level of methylation across the CpG dinucleotides in contrast to primary leukemia samples which show methylation index (MI) values ranging from highly methylated samples with MI of 62-97%, followed by samples showing less extensive methylation across the CG dinucleotides and samples that were classified as unmethylated.

### Gene expression and methylation status

**Figure 4:**
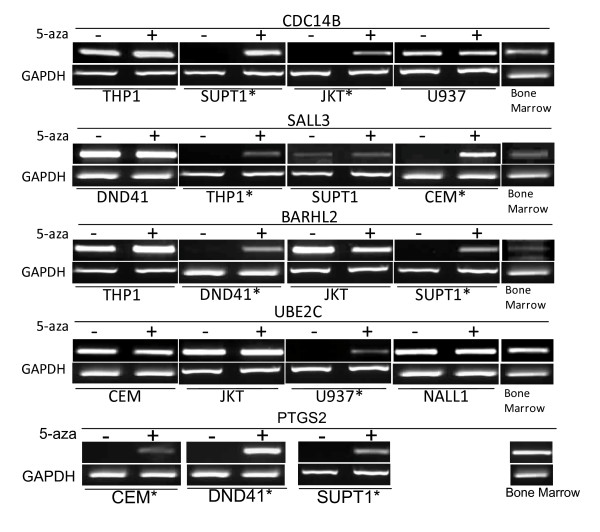
**Re-expression of Methylated genes**. Representative examples of leukemia cell line expression data. Expression from cell line RNA samples before demethylation treatment are labelled '-' and after demethylation treatment '+'. Methylated cell lines are labelled with a '*', expression of these genes in methylated cell lines before treatment could not be detected, expression in the same cell lines was detected after demethylation treatment. In unmethylated cell lines expression of these genes was detected before and after demethylation treatment to similar levels.

We demonstrated that genes listed in Table 1 (our positive genes) were expressed in control/normal bone marrow (Table 1; Figure 4). Leukemia cell lines were treated with 5-aza-2'-deoxycytidine (5azaDC) with or without Trichostatin A (TSA). We assayed expression of 26 of the 32 genes in leukemia cell lines before and after 5azaDC with or without TSA. All 26 genes showed restoration or upregulation of gene expression following the above treatment in methylated leukemia cell lines, whilst unmethylated cell lines showed similar expression levels before and after treatment.

### Functional pathway analysis of methylated genes

**Figure 5:**
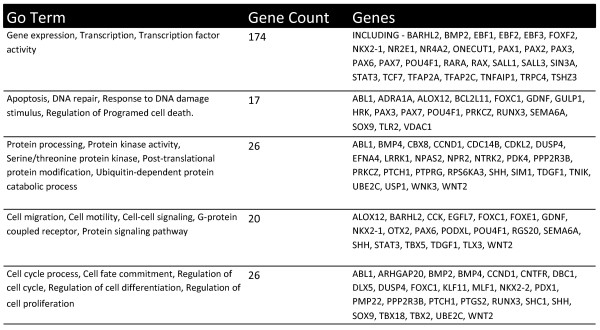
**DAVID functional annotation**. The table shows the results of using the online DAVID functional clustering and annotation program to cluster the short list of 398 genes in to functional groups and pathways. The analysis revealed that the majority of genes cluster as being involved in transcription, with smaller number of genes involved in functions such as apoptosis, DNA repair, cell cycle progression and cell signalling.

The resulting short-list of genes (n = 398) was functionally annotated using the DAVID bioinformatics tools [9]. Functional analysis revealed that by far the majority of the genes were involved in regulation of transcription including homeobox genes and transcription factors (Figure 5, Additional file 3). Since transcription factors and homeobox genes are known targets of the repressive polycomb complex in embryonic stem cells, we compared the above short list of genes with the list of genes marked as polycomb targets in human ES cells [10]. Of the 398 genes identified by MIRA, 45% were found to be polycomb targets, whilst amongst the validated genes shown to be regulated by promoter region methylation 44% were polycomb target genes. Other pathways represented included, apoptosis, DNA repair, protein processing/activity, cell migration, cell-cell signalling, regulation of cell cycle/cell differentiation and proliferation.

From the available clinical and genetic/cytogenetic data in this cohort of ALL samples we did not find any associations with gene methylation. This is likely due to the limited clinical-pathological data available for these samples. Hence we looked at another leukemia subtype in order to determine if gene methylation was related to any clinical-pathological features, see below for analysis in chronic myeloid leukemia.

### Gene methylation analysis in chronic myeloid leukemia

Unlike ALL, chronic myeloid leukemia (CML) has distinct disease progression stages. CML progresses from chronic phase to advance stage disease which includes a period of biological and clinical acceleration known as the accelerated phase and blast crisis [11]. We wanted to determine if our frequently methylated genes in ALL were also methylated in CML and if they played a role in CML biology. We analysed a cohort of CML patient DNA consisting of 55 samples from chronic phase CML (CP-CML) patients and 8 samples from blast crisis patients (BC), 5 of these CP and BC-CML were matched pairs, i.e. from the same patient at different stages of the disease. We demonstrated that only *ATG16L2 *showed frequent methylation in CP-CML samples (69%), whilst the rest of the genes were either unmethylated or demonstrated methylation frequencies below 20% in CP-CML patients. Hence *ATG16L2 *gene is frequently hypermethylated in both lymphoid and myeloid malignancies. In BC-CML samples 4 genes demonstrated frequencies of >25%, including *ATG16L2 *(63%). Amongst the remaining 3 genes, *TFAP2A *(63%) and *EBF2 *(57%), demonstrated statistically significant methylation in BC vs CP-CML (P < 0.05), whilst *TRCP4 *was more frequently methylated in BC (50%) compared to CB-CML (18%) samples but it did not reach statistical significant (P= 0.06) (Table 2). Four out of the 5 matched CP/BC patient samples demonstrated increased methylation in the DNA from BC phase as compared to the chronic phase DNA sample from the same patient, including methylation of the *TFAP2A*, *EBF2 *and *TRCP4 *genes. One matched patient sample became methylation positive for 10 additional genes on the transition from CP to BC.

**Table 2:** Genes showing differential methylation between chronic phase (CP) and blast crisis (BC) chronic myeloid leukemia (CML)

GENE	CP	BC
ATG16L2	34/49 (69%)	5/8 (63%)

TFAP2A	3/53 (6%)	5/8 (63%)

EBF2	6/52 (11.5%)	4/7 (57%)

TRCP4	10/55 (18%)	4/8 (50%)

Looking at major molecular response (MMR) to imatinib treatment, patients in whom *ATG16L2 *was methylated at baseline had an inferior chance of achieving MMR at 12 or 18 months, compared with the cases which were unmethylated at baseline. At 12 months only 1 of 14 methylated samples (at 18 months 3 of 12) demonstrated a significant molecular response, whilst amongst the 13 unmethylated cases 7 patients (at 18 months 10 of 13) demonstrated molecular response to imatinib therapy (p = 0.013 and 0.017 at 12 and 18 months respectively). The other genes methylation pattern at baseline did not predict the MMR or CCR (complete cytogenetic response) rates at 12, 18 or 24 months.

### Gene methylation analysis in common epithelial cancers

To determine if the frequently methylated genes in ALL samples could also be target of methylation in common epithelial cancers, we analysed 58 tumor cell lines consisting of colorectal (n = 10), lung (n = 14), breast (n = 9), kidney (n = 12), prostate (n = 5) and glioma (n = 8) for methylation. Ten genes (*BARHL2, EBF2, GPR123, NR2E1, PAX6, POU4F1, SALL3, TCF2, TFAP2A, TP53I11*) demonstrated methylation frequency of 50% or above in three or more tumor types (Table 3, see Additional file 4). Whilst further nine genes showed methylation frequency of 50% or above in at least one tumor type (*ATG16L2, CYP1B1, FOXF2, NKX2-1, NR4A2, PAX2, PRDM12, TRPC4, TSHZ3*). There were four genes (*CDC14B, FAT1, KNDC1, UBE2C*) that demonstrated no or very low level methylation across all epithelial tumor cell lines analysed.

**Table 3:** Summary of the promoter hypermethylation frequencies in epithelial cancer cell lines.

Gene	Colorectal	LUNG	BREAST	KIDNEY	GLIOMA	Prostate
ATG16L2	0/9	2/13	1/9	7/10	1/6	0/5

BARHL2*	4/4	3/9	0/3	4/4	ND	3/5

BMP2	4/9	2/14	3/9	0/11	2/7	1/5

CDC14B	0/9	0/14	0/8	0/11	0/7	0/5

CYP1B1	7/9	2/14	1/9	3/5	1/7	2/5

EBF2*	7/9	9/13	4/9	7/10	0/7	0/4

EYA2	2/5	1/11	1/9	1/8	2/6	1/5

FAT1	0/9	1/14	0/9	0/11	0/6	0/5

FOXF2	6/10	0/14	2/9	7/12	3/7	1/5

GPR123*	5/6	5/13	5/9	6/12	1/7	2/5

HLA-F	5/9	8/14	0/9	3/12	1/7	1/5

KNDC1	0/9	0/11	0/9	0/11	1/7	1/5

NKX2-1	3/8	0/12	0/1	2/4	0/1	1/4

NR2E1*	8/9	5/14	5/8	9/11	2/6	4/4

NR4A2	0/9	2/13	0/9	2/12	0/7	4/5

PAX2	3/5	3/10	1/6	1/9	0/5	0/5

PAX6*	6/8	11/12	8/9	7/11	3/7	ND

POU4F1*	6/7	6/13	4/8	7/9	5/7	3/5

PRDM12	3/4	0/4	0/0	6/6	0/0	ND

PTGS2	3/9	1/14	1/9	1/12	1/7	2/5

SALL3*	8/8	6/12	6/8	8/8	5/7	3/5

TCF2*	5/9	8/12	5/9	0/12	5/7	2/5

TFAP2A*	5/9	9/14	2/9	5/7	7/8	1/5

TFAP2C	0/8	3/13	0/9	2/11	1/6	0/5

TP53I11*	2/9	3/13	5/9	8/12	4/7	2/5

TRPC4	8/9	5/14	5/9	4/11	1/7	1/5

TSHZ3	6/7	4/13	3/7	2/11	0/7	ND

UBE2C	0/9	0/14	0/9	0/8	0/6	0/4

The table summarises all of the genes assessed for hypermethylation across cell lines from six epithelial cancers. The symbol * indicates genes showing methylation frequency of 50% or above in 3 or more epithelial cancer cell lines.

## Discussion

We have combined the use of genome-wide CpG island arrays with a novel and sensitive method, the methylated-CpG island recovery assay (MIRA) to identify frequently methylated genes in acute lymphoblastic leukemia and other cancers. Global profiling of a small number (n = 5) of samples yielded a large number of methylation targets that were validated in a large cohort of clinical samples. Hence the MIRA-based CpG island microarray platform proved to be both efficient and effective. We identified and validated 30 genes that were methylated in 25% or more of ALL samples and 2 genes that were methylated at a frequency of 23%. Amongst these genes, 19 were newly identified methylation targets in cancer, whilst the remaining genes had been shown to undergo methylation in other cancers but this is the first report for methylation in ALL. The validated genes fell into several major functional categories, including transcription factors (*TFAP2A, TFAP2C, EBF2, TCF2, PAX6, PAX2, FOXF2*), cell cycle control (*CDC14B, UBE2C*), phosphatases (*EYA2, DUSP4*), transforming growth factor-beta (TGFB) superfamily (*BMP2*). G protein-coupled receptor (*GPR123*), p53 gene target (*TP53I11*), homeobox (*TSHZ3, NKX2-1, BARHL2, POU4F1*), enzymes (*PTGS2*), ion channels (*TRPC4*), cadherin superfamily (*FAT1*), Zinc finger (*PRDM12*), nuclear receptor subfamily (*NR2E1*, *NR4A2*), ras association domain containing proteins (*MYO10*, *ARHGAP20*, *SSPN*). Forty-four percent of the validated genes (and 45% of the short list) were targets of the polycomb complex in embryonic stem cells. This percentage is similar to what has been reported in other cancers including lung, breast, colorectal cancer and follicular lymphoma[7,12-14]. Our data provides further evidence that polycomb group proteins have an impact on the epigenetic programming of gene expression in a wide range of cancers.

Amongst the validated genes, three genes have been shown to undergo genetic inactivation events in sub types of leukemia and proposed to act as tumor suppressor genes. *GPR123 *and *KNDC1 *have recently been shown to be mutated in acute myeloid leukemia by using whole-genome sequencing methodology [15], whilst *PRDM12 *is located in a minimal commonly deleted region in chronic myeloid leukemia[16].

A recent large-scale genome-wide study to identify genes methylated in adult ALL employing different high throughput approaches (MCA/RDA and MCA/array) validated 15 genes as showing frequent methylation in ALL (*GIPC2, RSPO1, MAGI1, CAST1, ADCY5, HSPA4L, OCLN, EFNA5, MSX2, GFPT2, GNA14, SALL1, MYO5B, ZNF382, MN1*) [17]. In our initial MIRA assay, we also identified 9 of the above 15 genes as frequent targets of methylation in childhood ALL, we did not analyze them any further since they had already been identified and validated in the Kuang *et al *study [17].

Chronic myeloid leukemia (CML) is a myeloproliferative neoplasm arising at the level of a pluripotent stem cell and consistently associated with the *BCR-ABL1 *fusion gene. CML most commonly manifests in a chronic phase of the disease that progresses to advanced stage disease (blast crisis) that is resistance to therapy [11]. Hence it is important to understand biological events involved in CML disease progression. We investigated the methylation status of the above genes (frequently methylated genes that were identified using the MIRA assay and validated in ALL samples) in a cohort of CML chronic phase and CML blast crisis samples. This led to the identification of two genes (*TFAP2A, EBF2*) that showed a significant increase in methylation in CML-BC compared to CML-CP samples. We further determined that in paired samples from the same patient, BC samples showed methylation of multiple genes (including the above 2 genes) whilst the corresponding CP samples were mostly unmethylated. Hence we have identified genes that are likely to play a role in CML disease progression. *TFAP2A*, a sequence specific DNA binding transcription factor has been demonstrated to be frequently methylated in large B-cell lymphoma, renal cell carcinoma and breast cancer [18-20]. *TFAP2A *has been shown to act as a tumor suppressor gene and plays an important role in cancer cell chemosensitivity. In breast cancer cells it has been demonstrated that expression of *TFAP2A *increased the chemosensitivity of cancer cells by sensitizing cells to undergo apoptosis upon chemotherapy[20]. Methylated breast cancer cell lines treated with 5-aza-2'-deoxycytidine induced reexpression of *TFAP2A*, resulted in apoptosis induction, increased chemosensitivity, decreased colony formation and loss of tumorigenesis upon chemotherapy. Amongst the 5 matched paired DNA samples (CML-CP and CML-BC from the same patient), *TFAP2A *was unmethylated in all 5 CML-CP samples but was methylated in 3 out of the 5 corresponding CML-BC samples. Hence *TFAP2A *may play an important role in CML-BC samples that become resistant to chemotherapy.

We also demonstrated that patients with methylation of *ATG16L2 *(ATG16 autophagy related 16-like 2), an autophagy related gene, had a significantly decreased rate of major molecular response (MMR, defined as a BCR-ABL: ABL transcript ratio of 0.1% or less) at 12 and 18 months of imatinib treatment in comparison with patients with unmethylated *ATG16L2 *gene (p = 0.013 and 0.017 at 12 and 18 months respectively). Other bona fide autophagy genes including *Beclin 1 *have been shown to act as tumor suppressor genes in cancer. *Beclin 1 *which is required for autophagy induction is monoallelically deleted in a high percentage of human breast, ovarian and prostate cancers, and its expression suppresses the tumorigenicity of human cancer cell lines [21-24]. It likely acts as a haploinsufficient tumor suppressor gene. Beclin 1 heterozygous deficient mice have decreased autophagy and spontaneously develop tumors [22]. Beclin 1 forms complexes with a range of proteins including UVRAG and Bif; these two proteins may also act as tumor suppressors [25,26]. UVRAG is frequently deleted (monoallelically) in colon cancers and overexpression leads to suppression of cell proliferation and tumorigenicity in human colon cancer cells [25]. Whilst deletion of Bif in mice results in the development of spontaneous tumors [26]. Recently ATG16L1 (ATG16 autophagy related 16-like 1) has been shown to be a bona fide autophagy protein [27]. Another autophagy inducing gene *DAPK-1 *is frequently silenced in human cancers by methylation and demonstrates tumor and metastasis suppressor properties [28]. Future studies should be aimed at analyzing a larger series of CML samples for *ATG16L2 *epigenetic inactivation and follow-up clinical parameters and at understanding the role it may play in CML development.

It would be very useful to identify epigenetic markers that could be utilized across several malignancies, to this end we determined the methylation status of the frequently methylated genes identified in ALL in six commonly occurring epithelial cancers (lung, breast, colorectal, kidney, brain and prostate). Ten genes (*BARHL2, EBF2, GPR123, NR2E1, PAX6, POU4F1, SALL3, TCF2, TFAP2A, TP53I11*) demonstrated methylation frequencies of 50% or higher in 3 or more epithelial cancers. Although our data is from methylation analysis of epithelial tumor cell lines, one would expect that some if not many of the above genes showing 50% or higher methylation frequency in tumor cell lines would also show frequent methylation in primary tumors. Our preliminary data for transcription factor *EBF2 *suggests that this indeed is the case (*EBF2 *is methylated in >25% of lung and breast tumors, data not shown). In addition, *PAX6 *methylation has previously been observed to occur frequently in colorectal and other cancers, *EYA2 *is methylated in colorectal cancer, *NKX2-1 *is methylated in thyroid cancer, *SALL3 *is methylated in hepatocellular carcinoma, *TCF2 *in ovarian cancer and *TFAP2A *is methylated in large B-cell lymphoma, breast and kidney cancers [18-20,29-33]. We carried out a limited analysis of DNA from normal/control epithelial tissues (brain, breast and kidney), these were found to be unmethylated for the genes that were frequently methylated in the corresponding cancers.

## Conclusion

In summary, we have identified and validated a large number of frequently methylated genes in ALL. In chronic myeloid leukemia epigenetic inactivation of 2 of these genes is associated with advanced disease and the hypermethylation of another gene is associated with poorer prognosis in CML patients. In addition a large number of these genes may also be frequently methylated in common epithelial cancers. Future studies of this group of genes may provide insights into pathogenesis of leukemia and other cancers and the identified methylation targets could be developed into molecular markers for early detection or therapeutic intervention across various cancers.

## Methods

### Patient samples

Twelve leukemia cell lines (DND-41, CCRF-CEM (CEM), U937, Jurkat (JKT), TALL-1, NALM1, NALM6, NALM16, NALM17, THP-1, SUP-T1 and MOLT-4) and 64 primary childhood ALL comprising 52 B-cell ALL (B-ALL) and 12 T-cell ALL (T-ALL) were analyzed. Characteristics of the ALL samples have been described previously and see additional file 5[4,5]. In addition *BCR-ABL *positive CML samples consisting of 55 chronic phase and 8 blast crisis samples (including 5 matched CP and BC samples from the same patient) were also analyzed. DNA from a total of 10 normal peripheral blood lymphocytes and normal bone marrow (BM, AMS Biotechnology) sample were used as controls. All DNA samples from patients were obtained with informed consent and followed institutional guidelines.

Nine colorectal cancer cell lines (174T, DLD1, HCT116, HT29, LOVO, LS411, SW48, SW60, SW480), fourteen Lung cancer cell lines (A549, H1155, H1299, H1395, H1437, H157, H187, H1648, H1792, H187, H1993, H2171, H 2122, H460), nine breast cancer cell lines (HCC1143, HCC1395, HCC1419, HCC1437, HCC1806, HTB29, MCF7, T47D, *MDA-MB-*231), seven Glioma cell lines (A172, H4, Hs683, T17, U87, U343, U373), twelve Renal cell carcinoma cell lines (768-O, CAKI, KTCL 26, KTCL 140, RCC4, SKRC18, SKRC39, SKRC45, SKRC47, SKRC54, UMRC2, UMRC3) and five Prostate cancer cell lines (22Rv1, DU-145, LNCaP, PC3 and VCap) were used in the Epithelial cancer cell line methylation panel. The collection of epithelial cancer cell lines have been described in our previous publications.

### Imatinib response criteria for CML

Response to imatinib treatment was defined conventionally [34]: complete cytogenetic response (CCR) = no Ph+ metaphases among at least 20 bone marrow metaphases or a BCR-ABL/ABL ratio of 1% or less; Major molecular response (MMR) = a BCR-ABL/ABL ratio of 0.1% or less.

### Detection of DNA methylation changes by MIRA-assisted microarray platforms

Genomic DNA was fragmented by sonication and MIRA binding reaction was set up on 200 ng of sonicated DNA as described previously [8]. The fraction representing the methylated DNA was collected from the binding reaction by Ni-NTA magnetic beads (Promega, Madison, WI) and washed 3 times with a 700-mM NaCl-containing buffer. Magnetic beads carrying the isolated fraction were picked up in 200 μl of TE buffer, mixed with one volume of phenol/chloroform and vortexed extensively. Magnetic beads were extracted by a magnet and the released methylated DNA fraction containing supernatant was ethanol precipitated after separation of the two phases in a microfuge. Isolated fraction was blunt-ended with T4 DNA polymerase (New England Biolabs), and a double-stranded adaptor was ligated onto the ends. Amplicons were created by LM-PCR. Labeling and array hybridization on human CpG island microarray platform (Agilent Technologies) were performed as described [8]. MIRA-enriched control and MIRA-enriched ALL amplicons were hybridized onto the CpG microarray for detection of ALL-specific methylation changes. Microarray slides were scanned using an Axon GenePix 4000b scanner and images wee quantified by GenePix Pro 6 software.

### Gene selection

The short list of 398 genes was generated as described in results section, also see figure 1.

### Methylation analysis

Bisulfite modification of DNA was performed as described previously [4]. The methylation status of all the CpG islands were determined by combined bisulfite restriction analysis (COBRA), semi-nested primers were designed to amplify regions of the CpG islands close to or overlapping the transcription start sites from bisulfite modified DNA (see additional file 6 for primer sequences). For enzymatic methylation detection ten microlitres of COBRA PCR product was incubated with 2U *BstUI *restriction enzyme (CGCG) overnight at 37°C before visualisation on a 2% agarose gel. The methylation status of *ARHGAP20, CDC14B, CYP1B1, EYA2, FAT1, GPR123, KNDC1, MYO10, PTGS2, SALL3, TFAP2A, TFAP2C *and *TRPC4 *was also determined by bisulfite sequencing. Samples selected for bisulfite sequencing were cloned in the pGEM-Teasy vector according to manufacturer's instructions. Up to 10 individual colonies were chosen for colony PCR using the primers specific for the pGEM-Teasy vector back bone, forward 5'-TAATACGACTCACTATAGGG-3' and reverse 5'-ACACTATAGAATACTCAAGC-3'. Amplified PCR products were then sequenced to ascertain the methylation status of individual alleles and to determine the methylation index (MI). The MI was calculated as a percentage using the equation; number of CpG dinucleotides methylated/total number of CpG dinucleotides sequenced × 100.

### Cell lines, 5azaDC treatment and RT-PCR

Leukemia cell lines were maintained in RPMI1640 (Sigma) supplemented with 10% FCS, 2 mM Glutamine, 20 mM HEPES, 1 mM Sodium Pyruvate and 12.6 mM Glucose Monohydrate at 37°C, 5% CO_2_. Cells were treated with 5 μM of the DNA demethylating agent 5azaDC (Sigma) freshly prepared in ddH_2_O and filter-sterilised. The medium (including 5 μM 5azaDC) was changed every day for 5 days. Cells were also treated on day 4 with 0.1 μM TSA for 24hrs. RNA was prepared using RNA bee (AMS biotechnology) according to manufacturers' instructions. cDNA was generated from 1 μg total RNA using SuperScript III (Invitrogen) and polyN primers. See additional file 7 for primer sequences. In all cases a *GAPDH *control was included using conditions described previously [4]. Gene expression was detected by amplification from 50 ng of cDNA using 0.8 μM of each primer, 2 mM MgCl_2_, 0.25 mM dNTPs and 1U Fast start *Taq *(Roche).

### Statistical analysis

Statistical analysis was performed using Fisher's exact test or *t *test where appropriate. All reported *P *values were two-sided and *P *< 0.05 was taken as statistically significant.

## Competing interests

The authors declare that they have no competing interests.

## Authors' contributions

FL designed research, did statistical analysis, established all collaborations and wrote the paper. TD did majority of the bioinformatics analysis, gene methylation, sequencing and expression analysis and took part in writing the paper. TR and GPP did the MIRA and array hybridization. LH took part in gene methylation, sequencing/expression analysis. DG did tissue culture. LW and RC provided the CML samples together with relevant clinical information and took part in statistical analysis. DC provided the ALL samples. ERM was involved in statistical analysis and in overall study design. AD took part in bioinformatics analysis. All authors reviewed the paper.

## Supplementary Material

Additional file 1 **COBRA analysis in leukemia cell lines**. Leukemia cell lines analyzed for methylation using COBRA. U = undigested PCR product, B = BstUI digested PCR product. Click here for file

Additional file 2 **additional genes**. genes that are not frequently methylated in ALL samples and/or are also methylated in control samples. Click here for file

Additional file 3 **Statistical analysis of the GO-terms used**. The P-values for all the GO-terms used in Figure 5 using David annotation Click here for file

Additional file 4 **COBRA analysis in epithelial cancer cell lines**. Methylation analysis in epithelial cancer cell lines. U = undigested PCR product, B = BstUI digested PCR product. The cell lines labelled with * correspond to either completely methylated or partially methylated cell lines. Click here for file

Additional file 5 **Patient characteristics**. Acute lymphoblastic leukemia patient characteristics Click here for file

Additional file 6 **COBRA primer sequences for the frequently methylated genes**. methylation primers used in this study Click here for file

Additional file 7 **Expression primer sequences for the frequently methylated genes**. expression primers used in this study Click here for file
