# Supplementary material for: A Genome-wide screen identifies frequently methylated genes in haematological and epithelial cancers
Source: Mol Cancer. 2010 Feb 25;9:44. doi: 10.1186/1476-4598-9-44 (PMC2838813; doi:10.1186/1476-4598-9-44)
Supplement: Additional file 1 — COBRA analysis in leukemia cell lines. Leukemia cell lines analyzed for methylation using COBRA. U = undigested PCR product, B = BstUI digested PCR product. [file 1476-4598-9-44-S1.PPT]

## Slide 1
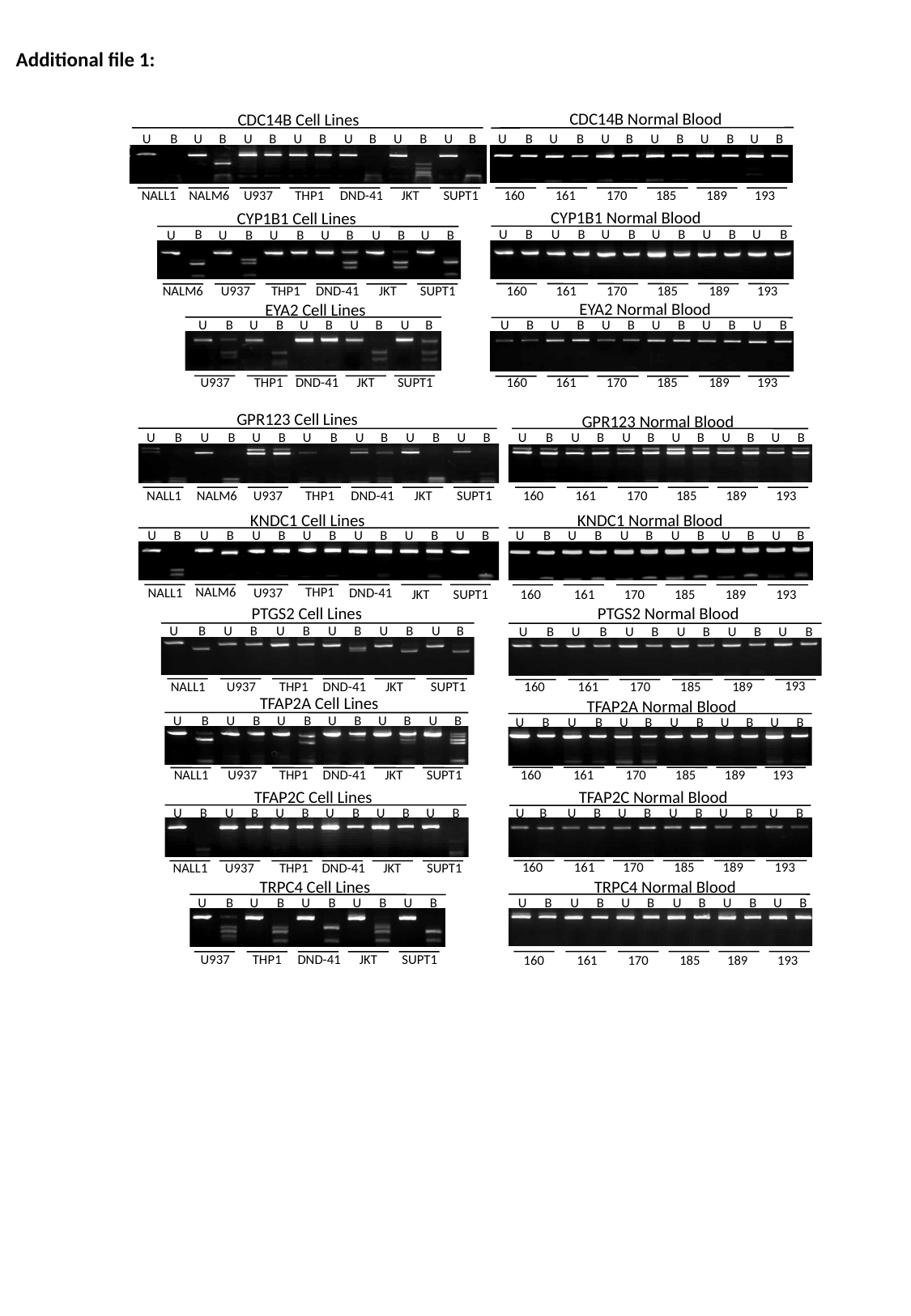

Additional file 1:
CDC14B Normal Blood
CDC14B Cell Lines
U
B
U
B
U
B
U
B
U
B
U
B
U
B
U
B
U
B
U
B
U
B
U
B
U
B
NALL1
NALM6
U937
THP1
DND-41
JKT
SUPT1
160
161
170
185
189
193
CYP1B1 Normal Blood
U
B
U
B
U
B
U
B
U
B
U
B
CYP1B1 Cell Lines
B
U
U
B
U
B
U
B
U
B
U
B
NALM6
U937
THP1
DND-41
JKT
SUPT1
160
161
170
185
189
193
EYA2 Normal Blood
U
B
U
B
U
B
U
B
U
B
U
B
160
161
170
185
189
193
EYA2 Cell Lines
U
B
U
B
U
B
U
B
U
B
U937
THP1
DND-41
JKT
SUPT1
GPR123 Cell Lines
U
B
U
B
U
B
U
B
U
B
U
B
U
B
NALL1
NALM6
U937
THP1
DND-41
JKT
SUPT1
GPR123 Normal Blood
U
B
U
B
U
B
U
B
U
B
U
B
160
161
170
185
189
193
KNDC1 Cell Lines
U
B
U
B
U
B
U
B
U
B
U
B
U
B
NALM6
THP1
NALL1
U937
DND-41
JKT
SUPT1
KNDC1 Normal Blood
U
B
U
B
U
B
U
B
U
B
U
B
160
161
170
185
189
193
PTGS2 Cell Lines
U
B
U
B
U
B
U
B
U
B
U
B
NALL1
U937
THP1
DND-41
JKT
SUPT1
PTGS2 Normal Blood
U
B
U
B
U
B
U
B
U
B
U
B
193
160
161
170
185
189
TFAP2A Cell Lines
U
B
U
B
U
B
U
B
U
B
U
B
NALL1
U937
THP1
DND-41
JKT
SUPT1
TFAP2A Normal Blood
U
B
U
B
U
B
U
B
U
B
U
B
160
161
170
185
189
193
TFAP2C Cell Lines
U
B
U
B
U
B
U
B
U
B
U
B
NALL1
U937
THP1
DND-41
JKT
SUPT1
TFAP2C Normal Blood
U
B
U
B
U
B
U
B
U
B
U
B
160
161
170
185
189
193
TRPC4 Normal Blood
U
B
U
B
U
B
U
B
U
B
U
B
160
161
170
185
189
193
TRPC4 Cell Lines
U
B
U
B
U
B
U
B
U
B
U937
THP1
DND-41
JKT
SUPT1
