# Supplementary material for: A Genome-wide screen identifies frequently methylated genes in haematological and epithelial cancers
Source: Mol Cancer. 2010 Feb 25;9:44. doi: 10.1186/1476-4598-9-44 (PMC2838813; doi:10.1186/1476-4598-9-44)
Supplement: Additional file 2 — additional genes. genes that are not frequently methylated in ALL samples and/or are also methylated in control samples. [file 1476-4598-9-44-S2.DOC]

| Gene | Methylation in cell lines | Notes |  |
| --- | --- | --- | --- |
| HLA-G | 4/6 | Methylated in healthy bone marrow |  |
| HMX2 | 3/6 | Same as above |  |
| PAX7 | 7/7 | Same as above |  |
| PAX9 | 7/7 | Same as above |  |
| TAC1 | 5/5 | Same as above |  |
| VSNL1 | 5/6 | Same as above |  |
|  |  |  |  |
| TLR2 | 7/8 | Not methylated in primary samples |  |
| TNFAIP1 | 7/9 | Same as above |  |
|  |  |  |  |
| CEP250 | 0/9 | Not methylated in leukemia cell lines |  |
| DAZAP2 | 0/7 | Same as above |  |
| MYO9B | 0/8 | Same as above |  |
| ONECUT | 0/6 | Same as above |  |
| RAPGEF6 | 1/10 | Same as above |  |
| RARA | 0/6 | Same as above |  |
| RAX | 0/6 | Same as above |  |
| RGL2 | 0/7 | Same as above |  |
| SIN3A | 0/6 | Same as above |  |
| SEL1L | 0/7 | Same as above |  |
| STAT3 | 0/7 | Same as above |  |
| TANK | 0/5 | Same as above |  |
| TCF7 | 0/6 | Same as above |  |
| USP1 | 0/7 | Same as above |  |

**Additional file 2**
