# Supplementary material for: A Genome-wide screen identifies frequently methylated genes in haematological and epithelial cancers
Source: Mol Cancer. 2010 Feb 25;9:44. doi: 10.1186/1476-4598-9-44 (PMC2838813; doi:10.1186/1476-4598-9-44)
Supplement: Additional file 3 — Statistical analysis of the GO-terms used. The P-values for all the GO-terms used in Figure 5 using David annotation [file 1476-4598-9-44-S3.DOC]

**Additional file 3**

Gene expression - 9.9E-29

Transcription - 1.8E-29

Transcription factor activity - 2.7E-54

----

Apoptosis - 3.5E-1

DNA Repair - 9.8E-1

Response to DNA damage stimulus - 9.9E-1

Regulation of programmed cell death - 3.0E-1

---

Protein processing - 6.4E-1

Protein kinase activity - 5.2E-1

Serine/threonine protein kinase - 7.5E-1

Post-translational protein modification - 9.9E-1

Ubiquitin-dependent protein catabolic process - 9.2E-1

---

Cell migration - 2.1E-6

Cell motility - 6.6E-2

Cell-cell signaling - 3.9E-7

G-protein coupled receptor protein signaling pathway - 7.0E-1

---

Cell cycle process - 5.7E-1

Cell fate commitment - 7.5E-10

Regulation of cell cycle - 3.7E-1

Regulation of cell differentiation - 1.9E-4

Regulation of cell proliferation - 9.6E-5
