# Supplementary material for: A Genome-wide screen identifies frequently methylated genes in haematological and epithelial cancers
Source: Mol Cancer. 2010 Feb 25;9:44. doi: 10.1186/1476-4598-9-44 (PMC2838813; doi:10.1186/1476-4598-9-44)
Supplement: Additional file 4 — COBRA analysis in epithelial cancer cell lines. Methylation analysis in epithelial cancer cell lines. U = undigested PCR product, B = BstUI digested PCR product. The cell lines labelled with * correspond to either completely methylated or partially methylated cell lines. [file 1476-4598-9-44-S4.PPT]

## Slide 1
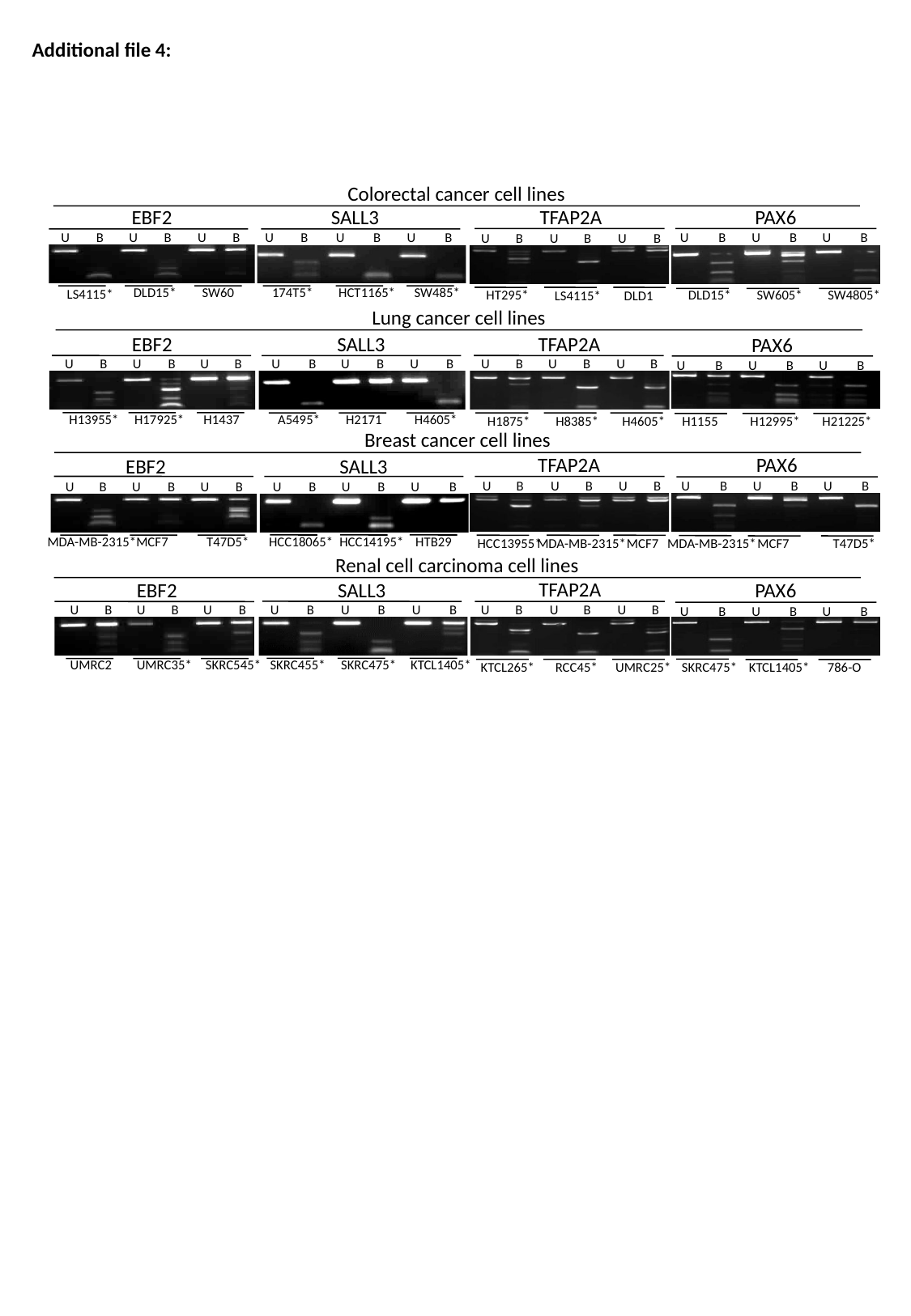

Additional file 4:
Colorectal cancer cell lines
EBF2
SALL3
TFAP2A
PAX6
U
B
U
B
U
B
U
B
U
B
U
B
U
B
U
B
U
B
U
B
U
B
U
B
HT295*
LS4115*
DLD1
DLD15*
SW60
174T5*
HCT1165*
SW485*
LS4115*
DLD15*
SW605*
SW4805*
Lung cancer cell lines
EBF2
U
B
U
B
U
B
H13955*
H17925*
H1437
TFAP2A
U
B
U
B
U
B
H1875*
H8385*
H4605*
SALL3
PAX6
U
B
U
B
U
B
U
B
U
B
U
B
A5495*
H2171
H4605*
H1155
H12995*
H21225*
Breast cancer cell lines
PAX6
TFAP2A
U
B
U
B
U
B
HCC13955*
MDA-MB-2315*
MCF7
EBF2
U
B
U
B
U
B
MDA-MB-2315*
MCF7
T47D5*
SALL3
U
B
U
B
U
B
U
B
U
B
U
B
HCC18065*
HCC14195*
HTB29
MDA-MB-2315*
MCF7
T47D5*
Renal cell carcinoma cell lines
TFAP2A
U
B
U
B
U
B
KTCL265*
RCC45*
UMRC25*
PAX6
EBF2
U
B
U
B
U
B
UMRC2
UMRC35*
SKRC545*
SALL3
U
B
U
B
U
B
U
B
U
B
U
B
SKRC455*
SKRC475*
KTCL1405*
SKRC475*
KTCL1405*
786-O
