# Supplementary material for: A Genome-wide screen identifies frequently methylated genes in haematological and epithelial cancers
Source: Mol Cancer. 2010 Feb 25;9:44. doi: 10.1186/1476-4598-9-44 (PMC2838813; doi:10.1186/1476-4598-9-44)
Supplement: Additional file 5 — Patient characteristics. Acute lymphoblastic leukemia patient characteristics [file 1476-4598-9-44-S5.DOC]

Additional file 5

B-cell 52

T-cell 36

Median age at diagnosis:

6.13 years (range 0.58-15.14 years)

Male sex 60

Female sex 27

Cytogenetics:

Hyperdiploid 23

Normal 46

TEL/AML rearrangement

Negative 41

Positive 15

PH negative 46

Complete remission 62

Relapse 8

Median Disease free survival 185 weeks

Median overall survival 194 weeks
