# Supplementary material for: A Genome-wide screen identifies frequently methylated genes in haematological and epithelial cancers
Source: Mol Cancer. 2010 Feb 25;9:44. doi: 10.1186/1476-4598-9-44 (PMC2838813; doi:10.1186/1476-4598-9-44)
Supplement: Additional file 6 — COBRA primer sequences for the frequently methylated genes. methylation primers used in this study [file 1476-4598-9-44-S6.DOC]

| Gene | Forward Primers (5’-3’) | Reverse Primers (5’-3’) | Tm (°C) | Size (bp) |
| --- | --- | --- | --- | --- |
| ARHGAP20 | F - AGGTTAGYGTTYGGGATTGTATAAGAGGTT | R – ACCTTTTCRATCTATAACTCCRCRTAAATACCA | 1 – 58 | 372 |
|  | FN - TAGTTYGTTGAAAGYGAGGTAGGAATTATTGT |  | 2 - 58 | 249 |
| ATG16L2 US | F - GTTTTTGTTTATGATTTTYGTAGTTATAGGAGT | R – CTCRTCCAAATTCTAACCAAAACCTAAATA | 1 - 57 | 586 |
|  |  | RN - TAACRCACAAACTCAAAACATAAAACACAA | 2 - 57 | 522 |
| BMP2 | F - ATTTTTAGYGTYGYGATGYGGAGTATT | R - CCTACAAATTCAAAAAATCCCCAACCAAAATA | 1 - 57 | 572 |
|  | FN - GAGGTGTGATYGGATTTTAGGTTAGTTAT |  | 2 - 57 | 540 |
| CDC14B US | F - GTTAGTATATTTTATTTTTGATTGGYGGAAGTT | R – RACCCRTCAAAAAACCACRACCATAA | 1 - 57 | 616 |
|  | FN - YGTYGTATTGGTTAATGAAAGGTTTTAGTT |  | 2 - 57 | 517 |
| CYP1B1 | F - GGAAAYGTGGGTATTTTYGTTTTTATGAAA | R - AAAAACCCRCAACCCRACCTAAACACCTA | 1 - 57 | 484 |
|  |  | RN - ACTAAAAACTTTAACTCCCACTCRAATCTCTTA | 2 - 57 | 477 |
| DUSP4 | F - GTTTTATGAATGGGTTTGATGGYGAGTAT | R - CCRCAACCTCRCRATCACATAACAA | 1 - 54 | 534 |
|  | FN - GGAGTTAGYGGTYGYGTTTTATTTAAGTTT |  | 2 - 54 | 456 |
| EBF2 | F - TTTGTTGGTAAYGTTGGGAATAGTTAGGAT | R – AAAAAACRCCTACRACCCAAACAATTCAAA | 1 - 57 | 445 |
|  |  | RN - AACRTTCRCCTAACTACAACRATCTACTTA | 2 - 57 | 361 |
| EYA2 | F – AGGAGGTTGGGTTTTGGTTTTTAGGAT | R - AACTCTACTACTATCCCCTCTACCCTTATA | 1 - 59 | 600 |
|  | FN - GTYGGTTTTTTYGGYGTAGGTAGTAGT |  | 2 - 59 | 367 |
| FAT1 | F - GTGGTTGGTYGTAATTAATTTYGGTATTATAAGTT | R – CCCCTAACCAATACTAAATTTCCCAACTAA | 1 - 57 | 650 |
|  | FN - TTTTAGTAYGGATTTGGTGTTYGTAGGGAT |  | 2 - 57 | 565 |
| FOXF2 | F - TTTYGGGGTTTAGGTYGYGGTTTTAT | R - CTCRATAATCATCTAAAACCCRAAAACRAAA | 1 - 55 | 345 |
|  |  | RN - ACRAAAACRCCRAAACRAAATCRACCCAAAAA | 2 - 55 | 283 |
| GPR123 ds | F – GYGGTYGTTYGGTTGGAAGAGTTATT | R - ACCRCRACRCCTACTCTACCTAAATATA | 1 - 58 | 565 |
|  |  | RN - TACTCRAAACTACTTTCCTCRCRACCAATA | 2 - 58 | 458 |
| HLA-F | F - GAAGATTTAGGGAGATATTGAGATATATTTTGTAT | R – CTCCCCACAAAAACCRTTTCTCTCTAA | 1 - 59 | 451 |
|  | FN - GTTTTAGGAGTGGTTTTTAAGGGTTTAGGT |  | 2 - 59 | 379 |
| KNDC1 US | F - TAGAGGTTATTTYGGGATTGYGGTAGT | R – CRACCRAATCCATAACCTACATCCTA | 1 - 59 | 475 |
|  | FN - TAGATTTYGTGGTTTTTYGYGGAGGTTAT |  | 2 - 59 | 331 |
| MOY10 | F - TTTTTAYGTTTAYGTGGGGAAGATTTGGGGAAAT | R – TTCTTCCTCCAAATTCCTCACTACTAA | 1 - 56 | 565 |
|  |  | RN - ACRCAACRCACRCAAACRAAAAACAACTAA | 2 - 58 | 538 |
| NKX2-1 | F – TGGTTYGGGGATTYGGGATAGTTTT | R – CCTACATCTTACCCRAAATAATTAACTTACATA | 1 - 58 | 736 |
|  | FN - TTTGAYGGYGGTAGAAGAGAGGTAGAT |  | 2 - 58 | 580 |
| NR2E1 | F - TTGGGAGTYGTTTGGAGGTTTATTAATATT | R – AAATAAAAACACRAAAATAACCTTCCTACRAAA | 1 - 57 | 534 |
|  |  | RN - RATCTCTCCCTCTACATAAACATACAAAATA | 2 - 57 | 461 |
| NR4A2 | F - TTTGTTYGTGAYGTTAGGTYGGAAATATAT | R – CRAACTACATAAACTACATCTACTAACTTAA | 1 - 58 | 414 |
|  |  | RN - AACTCTACCRAAATACAATTCCCTCTAA | 2 - 58 | 354 |
| PAX2 | F - AAGTTYGGGGTTTTAGYGTTGGYGAATTAT | R - RACTCRCTCAACACCCCTAAAACCTAA | 1 - 59 | 651 |
|  |  | RN - RACTCRCTCAACACCCCTAAAACCTAA | 2 - 59 | 483 |
| PAX6 US | F - AATTAGTYGGYGTAGAGTTGTGTTTAATTT | R – TTCRCRATCRCRAATAACTTTAACAAACAA | 1 - 57 | 606 |
|  |  | RN - CAAAACCRAACTAAAAATCCACTCTTAAAAATA | 2 - 57 | 535 |
| POU4F1 | F - AAAGGTGTTTATTTTTAGATAATAGTTGGATT | R - CATAACAAAATAAAACTACTTACTATTCATAA | 1 - 53 | 614 |
|  | FN - TATGTTY**G**GGTAGTTATAGGTGTATTATT |  | 2 - 53 | 535 |
| PRDM12 | F - AAGGTTTTTYGAGTTTAGTYGGGTATAAT | R – TTACRCCAACRACCRTACAAAAAACTA | 1 - 57 | 630 |
|  |  | RN - RAAAATAATAACCTCRACCAACRCCAT | 2 - 57 | 592 |
| PTGS2 | F - GGGTAAAGATTGYGAAGAAGAAAAGATATT | R – CRACRCCAAATACTCACCTATATAACTA | 1 - 57 | 524 |
|  | FN - GGGATTATTTTTTTTGTTTTTAAATTGGGGTAGT |  | 2 - 57 | 426 |
| SALL3 | F - TYGTATTYGGGTTTYGTTATAGTYGTATT | R - CCTCRTCCRACTTAAAATACTAAAACTTAA | 1 - 53 | 432 |
|  |  | RN - AACCRCRCATAAAACTAAACAATTAAACTAATA | 2 - 53 | 163 |
| SSPN | F – ATTAAAATAATTTTGGAGGTTTTGAGTGAGGAAAAT | R - CCRCRTAACTACTTATTCTTACCCATTA | 1 - 57 | 409 |
|  | FN - GTAGAGGTTTAGGYGGTTAAGGGAT |  | 2 - 57 | 337 |
| TACF2 | F - TAYGTTTTTTGGYGGGTAGTTTTGTAAATT | R – CTAAAAACCTTCTTTTACTAATTCTACTTATCAA | 1 - 57 | 463 |
|  |  | RN - CCAAACTTCACCTAACCTTTAAACTTATTAAA | 2 - 57 | 429 |
| TFAP2A L | F - GTTTTTATTTATTGAGTAATAGTTTTATGTGGAATT | R – CRCCRCACAAAACTAACTAAAAAATA | 1 - 57 | 478 |
|  | FN - GGTAGAGTTAGATTYGTTAAGGTTAGT |  | 2 - 57 | 428 |
| TFAP2C | F - ATGYGTAGTTTTTTAATTTTAGGTGATATT | R – ATCCAAAATCRAAATCACRATAACCAAA | 1 - 54 | 568 |
|  | FN - GTTGTTAAYGAGGTATYGTTTATTGAT |  | 2 - 54 | 382 |
| TP53I11 | F - AGGTATYGGTTTGGGTTYGGGAGATAT | R – RCRTACCRACRCTAAACTACRTAAAACTAATA | 1 - 59 | 573 |
|  | FN - GGTATYGTTTGTAGAATTTGTTAGGAAAGTTATT |  | 2 - 59 | 488 |
| TRPC4 | F – TTGGGTGTYGGAGYGTATTGAAGTTTAAT | R - CTCTAAATAAAATTAAAAATTCCCACRACTAACAA | 1 - 58 | 415 |
|  | FN - GGAGGTGTGTTTGGTTTGTATTTAGAATTAT |  | 2 - 58 | 360 |
| TSZH3 | F - GGAGYGATTTGTTAAATTTTTTATTTYGTYGGA | R – CTACTACRCRCCRAAACRCCTACTA | 1 - 59 | 389 |
|  |  | RN - ACATAATACTTCTCCRACRACYACCACTA | 2 - 59 | 348 |
| UBE2C | F - TTTTAATGGTTAGYGTTTTTTAAYGGTTAT | R - TTTACTCTAAATACCAATACCTAAAACATA | 1 - 55 | 476 |
|  | FN - GGATTYGTTAGTTAATGGTAGTATTATTTAT | TATGTTTTAGGTATTGGTATTTAGAGTAAA | 2 - 55 | 435 |

**Additional file 6**
