# Supplementary material for: A Genome-wide screen identifies frequently methylated genes in haematological and epithelial cancers
Source: Mol Cancer. 2010 Feb 25;9:44. doi: 10.1186/1476-4598-9-44 (PMC2838813; doi:10.1186/1476-4598-9-44)
Supplement: Additional file 7 — Expression primer sequences for the frequently methylated genes. expression primers used in this study [file 1476-4598-9-44-S7.DOC]

| Gene | Forawrd Primers (5’-3’) | Reverse Primers (5’-3’) | Tm(°C) | Size (bp) |
| --- | --- | --- | --- | --- |
| ARHGAP20 | TCTCTTATCCTGGATAAAGCC | TTCATCCACACAGCTTGCTG | 54 | 280 |
| ATG16L2 | GAGGAGTCAGAGCTTGACTCAG | TGCTTTGCCTCTGCTGCAGC | 57 | 172 |
| BMP2 | ACCTGTATCGCAGGCACTCAG | ACCTGAAGCTCTGCTGAGGTG | 59 | 214 |
| CDC14B | GACGTGTACCTGGACATCAC | CATGTAGCATCCAACAAGGAAG | 56 | 282 |
| CYP1B1 | CCACTATCACTGACATCTTCG | GTCATGATTCACAGACCACTG | 56 | 326 |
| DUSP4 | GTCAACGTGCGCTGTAACAC | GAACAGAATTCTGGGTACTCGG | 57 | 284 |
| EBF2 | TCCTGACGCACGAATGTATG | GACGTGTCCATCCACATTACA | 59 | 215 |
| EYA2 | AACGGCAGAGACAGCAACGTG | TGTCTGTCACTCAGAGTCCACAC | 59 | 226 |
| FAT1 | GTTGATGTGAATGAGAACCTGC | TCCAGTCGATCTGACGTCAC | 57 | 227 |
| FOXF2 | TGGAGCAGAGCTACTTGCAC | TTAATATCCTGACAGACGCTCTG | 57 | 193 |
| GRP123 | CATGCTGTGGATAGGAGTGAC | CGTAATTCCTGATGTTCGTGGC | 57 | 184 |
| HLA-F | ACATGCCATGTGCAGCACGAC | CTGCAGCCTGAGAGTAGCTC | 58 | 202 |
| KNDC1 | AGCGACAGCCTGTGTCTGATG | GGTGTAAGGGTTCTCCTGGAAC | 59 | 202 |
| MYO10 | GGTCTGGCTGAGAGAAAATG | TGATTTCTCTTATACCGCTGG | 54 | 232 |
| NKX2-1 | GGGCAACATGAGCGAGCTGC | GGCCATGTTCTTGCTCACGTC | 60 | 190 |
| NR2E1 | CTACACATAGTGGTTCTGAAC | CTTGTGAGCTTAGATATCACTG | 54 | 275 |
| NR4A2 | TGTGAGGGCTGCAAAGGCTTC | GAGGGCACTGATCAGACTCAC | 59 | 226 |
| PAX2 | CAAAGTGGTGGACAAGATTGCTG | CAGTGGGATCCTACTCCATCA | 57 | 281 |
| PAX6 | CGGTGAGAAGTGTGGGAAC | CTGGTTGTCACAGCTTCTGTC | 57 | 232 |
| POU4F1 | ATGAACAGCAAGCAGCCTCAC | GCACGCTGTTCATCGTGTG | 57 | 237 |
| PRDM12 | TTCTCCGGCGAAGTGCAGAAG | CACCGTGCCATCCTCATTGAAC | 59 | 225 |
| PTGS2 | CCAGTATAAGTGCGATTGTAC | AGCCATAGTCAGCATTGTAAG | 54 | 254 |
| SALL3 | ATCCATGAGCGCACGCAC | CTGGAACATTTCAGAGAACTTCAG | 57 | 204 |
| SSPN | GTCTCATATCAGGTTGACGAAC | AGTGCCAGTGACGCTGGTAC | 56 | 202 |
| TCF2 | TGAGGTCCGTGTCTACAACTG | CTGTTGCCATGGTGACTGATTG | 56 | 240 |
| TFAP2A | CCCAGAGAGTAGCTCCACTTG | GAAGGGAGATTGACCTACAGTGC | 58 | 197 |
| TFAP2C | GACAGTGGCTGAAGTACAGAG | TCAGGAGAGTCACATGAGCG | 57 | 182 |
| TP53I11 | GCTGTGATTAGAGAAGCCAG | ATCTTGGAGCGATGCACCTC | 57 | 274 |
| TRPC4 | CAGCAGCGACTAAGGGAATTC | CTGATTCTGCTCTTACTATCC | 54 | 226 |
| TSZH3 | ATCATGCCGAGGAGGAAGCAG | CGGTCACTCGTCTCACTGATG | 59 | 254 |
| UBE2C | TTGAACACACATGCTGCCGAG | GCTCAAGATAAAGAGTCCTATACAG | 57 | 196 |

**Additional file 7**
